# Bouncing behavior of sub-four minute milers

**DOI:** 10.1038/s41598-021-89858-1

**Published:** 2021-05-18

**Authors:** Geoffrey T. Burns, Richard Gonzalez, Jessica M. Zendler, Ronald F. Zernicke

**Affiliations:** 1 School of Kinesiology, University of Michigan, 1402 Washington Heights, Ann Arbor, MI 48109-2013 USA; 2 Department of Psychology, University of Michigan, 004 East Hall, 530 Church Street, Ann Arbor, MI 48109-1043 USA; 3 Department of Orthopaedic Surgery, University of Michigan, 1500 E. Medical Center Drive 2912 Taubman Center, Ann Arbor, MI 48109-5328 USA; 4 Department of Biomedical Engineering, University of Michigan, 2200 Bonisteel, Blvd., Ann Arbor, MI 48109-2099 USA; 5 School of Kinesiology, University of Michigan, 401 Washtenaw Avenue, Ann Arbor, MI 48109-2214 USA

**Keywords:** Bone quality and biomechanics, Biomechanics

## Abstract

Elite middle distance runners present as a unique population in which to explore biomechanical phenomena in relation to running speed, as their training and racing spans a broad spectrum of paces. However, there have been no comprehensive investigations of running mechanics across speeds within this population. Here, we used the spring-mass model of running to explore global mechanical behavior across speeds in these runners. Ten elite-level 1500 m and mile runners (mean 1500 m best: 3:37.3 ± 3.6 s; mile: 3:54.6 ± 3.9 s) and ten highly trained 1500 m and mile runners (mean 1500 m best: 4:07.6 ± 3.7 s; mile: 4:27.4 ± 4.1 s) ran on a treadmill at 10 speeds where temporal measures were recorded. Spatiotemporal and spring-mass characteristics and their corresponding variation were calculated within and across speeds. All spatiotemporal measures changed with speed in both groups, but the changes were less substantial in the elites. The elite runners ran with greater approximated vertical forces (+ 0.16 BW) and steeper impact angles (+ 3.1°) across speeds. Moreover, the elites ran with greater leg and vertical stiffnesses (+ 2.1 kN/m and + 3.6 kN/m) across speeds. Neither group changed leg stiffness with increasing speeds, but both groups increased vertical stiffness (1.6 kN/m per km/h), and the elite runners more so (further + 0.4 kN/m per km/h). The elite runners also demonstrated lower variability in their spatiotemporal behavior across speeds. Together, these findings suggested that elite middle distance runners may have distinct global mechanical patterns across running speeds, where they behave as stiffer, less variable spring-mass systems compared to highly trained, but sub-elite counterparts.

## Introduction

The flight phase and the single-support stance phase that define running allow humans to realize an enormous variety of velocities, and no athletes are more fluent across this spectrum of speed than those of the middle distance runners. In one of the earliest studies examining the oxygen cost of running across speeds, Sargent chose his lone subject to be a competitive middle distance runner, as he was “a performer capable in both sprint and long-distance work”^1^. Their racing demands enormous fluctuations in speed, spanning the aerobic and anaerobic continuum^2^. At a global level, recent championships in the men’s 1500 m have seen average speeds ranging from 6.5 to 7.1 m/s^3^, with inter-lap variability often exceeding 10% of that^4^, and intra-lap variability even more^5^. This variety in competition itself requires even greater variety in training. The Australian 1500 m runner Herb Elliott, 1960 Olympic champion and former world record holder in the event, included in his training regimen not only runs themselves ranging from 220-yard maximal sprints to 30-mile distance sessions, but also “fast climbing of mountainsides, running up stairs of buildings up to ten stories, trudging in snow, and… long distance swimming”^6^. While modern middle distance training is slightly less eccentric, it still routinely consists of running that may span 50 to 115% of racing speeds with substantial volumes at the lower end of that spectrum^2,7,8^.

Middle distance runners therefore present as a population that have developed their gait patterns across a variety of speeds, and elite runners further present as a population that has refined their gait patterns under high volumes of training and substantial competitive pressure to maximize their performance capacities. They are therefore thought to have undergone a process of mechanical “self-optimization”^9,10^. Previous biomechanical investigations of elite distance runners have focused on middle-long and long distance specialists^11–14^, but relatively little work has examined the biomechanical patterns in elite middle distance runners. Leskinen and colleagues studied the in-race biomechanics of elite and national-class 1500 m runners (average bests: 3:36 and 3:49, respectively) via high-speed video. While the racers ran with similar speeds and contact times in the races studied, they observed the elite cohort to run with less knee flexion and greater knee stiffness during stance. They concluded that this may have indicated a more efficient recycling of energy through the gait cycle via elastic storage and return as opposed to the greater amount of concentric work observed in the lower-caliber runners^15^. Trowell and colleagues studied the kinematics and kinetics of a group ranging from regional-class to elite-level middle distance runners (best 1500 m: 3:31 to 4:01) at a racing speed (7.2 m/s), and they used multiple regression to identify mechanical characteristics that explained differences in performance. Contrary to the in-race findings of Leskinen and colleagues, they observed that the better runners exhibited greater knee flexion during stance as well as other subtle kinematic differences at the hip and ankle^16^. It is unclear if the differences in the findings were related to the methodology, context, or relative homogeneity of populations. Moreover, the studies were limited to single racing speeds. While this provides important insights pertaining to competition-specific patterns, it has yet to be established how these high-level runners do or do not alter their gait across the spectrum of speeds to which they are exposed and practiced.

**Figure 1:**
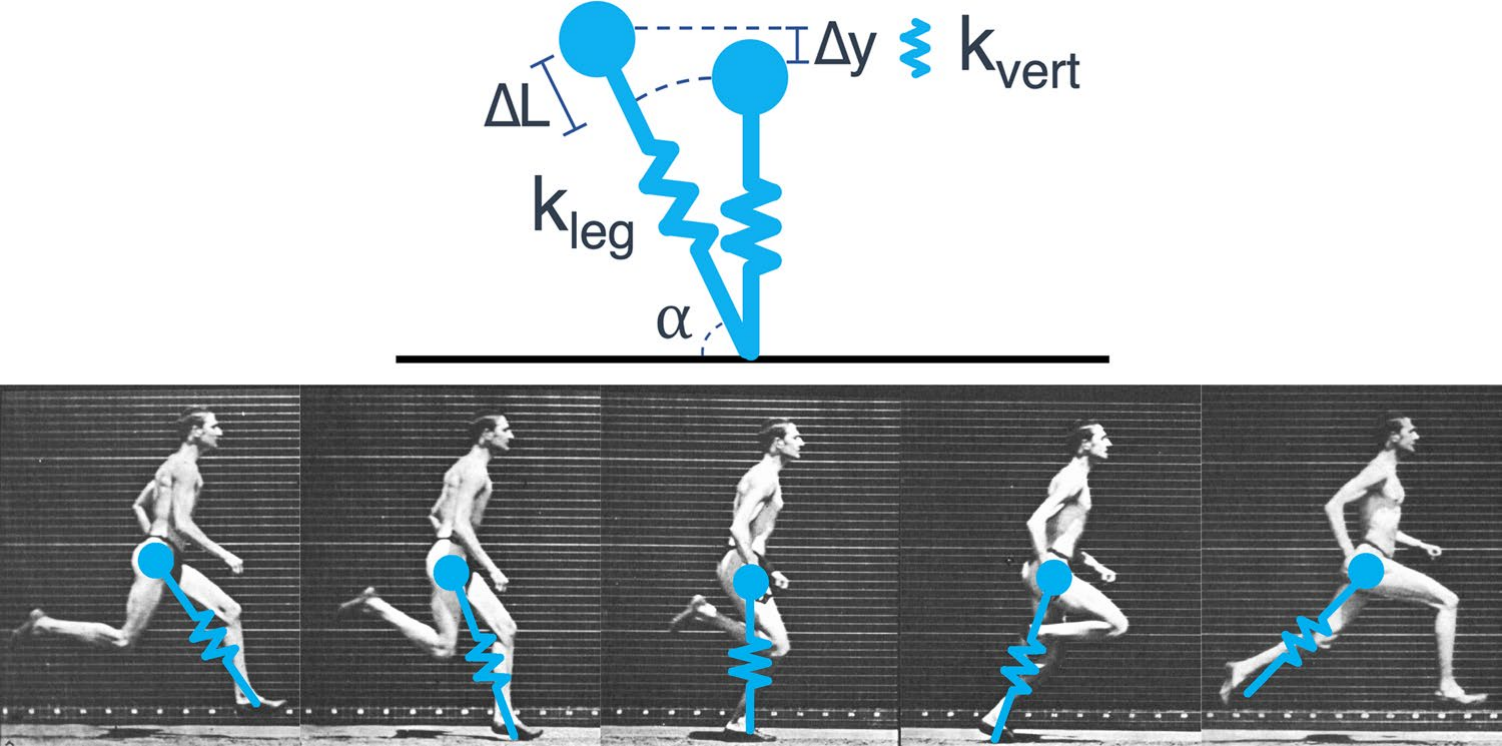
The spring-mass model of running (frames adapted from E. Muybridge^66^).

An alternative method to the component-level kinematic investigations described above is to assess the systemic characteristics of the runners. The spring-mass model is commonly used to describe runners as such^17,18^. It treats the runner as a single point-mass on a linear elastic spring that strikes and leaves the ground at a constant touchdown angle, and it has been proposed as the mechanical template that underlies the running gait across species^19^. Figure 1 shows the spring-mass model as it is interpreted in human running.

The model and its associated spatiotemporal characteristics have exhibited relations to running performance and economy, such as leg and vertical stiffness^20,21^, vertical oscillation^10,22,23^, contact time^24^, duty factor^23^, stride frequency^25^, and stride length^13^. It has been applied to elite runners in the study of sprinting^26^ and triathlon^27^, but it has not been systematically examined in elite middle distance runners. Rogers and colleagues studied leg stiffness in a group of highly-trained middle distance runners (average 1500 m best: 4:02) at a single submaximal speed (14 km/h) and at a maximal sprinting speed and observed that the leg stiffness in sprinting was strongly correlated to both running economy and maximal sprinting speed^28^. Similarly, Fourchet and colleagues studied the spring-mass characteristics of youth middle distance runners during an exhaustive run and found contact time, vertical displacement, and leg compression to increase following the run, resulting in decreased leg stiffnesses with consistent stride lengths and frequencies^29^. These investigations suggest the importance of spring-mass characteristics in mediating middle distance performance. Furthermore, the degree to which these characteristics are individualized within runners may have important implications for their performance and health. It has been postulated that individuals have an optimality with respect to lower limb stiffness and their own musculoskeletal system, where spring-mass behavior that is too stiff may subject the athlete to forces and loading rates that are too severe, while a system that is too compliant may predispose the athlete to soft-tissue injury^30^. Additionally, some spring-mass characteristics are speed dependent^17,31^, and while several studies have assessed these changes in runners of varying abilities^14,32,33^, none have systemically examined speed-dependent patterns in elite middle- or long-distance runners.

These model characteristics are also often reported as stationary values without indication of intra-individual variability patterns. Mechanical patterns within runners are not stationary^34–36^, and the patterns of variability may have implications for injury and performance^37,38^. It has been proposed that there are two broad classes of variability in biomechanical patterns: task-level outcome variability (e.g., stride length in running) and process-level component variability (e.g., joint coordination patterns). These are referred to as end-point variability and coordinative variability, respectively^37^. There are divergent thoughts on how coordinative variability relates to expertise in biomechanics, with some observing U-shaped curve for the amount of component variability^39^, some finding decreasing variability in important movements among skilled performers^40^, and some finding no difference between skill levels^41^. At the task level, it has been shown that variability decreases in more experienced or skilled performers in race-walking^42^ and running^43^. Belli and colleagues observed variability in center-of-mass displacement and step time to increase with running speed in moderately trained runners, with the amount of variability across speeds further bearing a moderate correlation to the energy cost of running^44^. Similarly, Candau and colleagues found a significant relation between lower levels of step frequency variability and better running economy and that the variability increased with fatigue^45^. Furthermore, outcome variability assessed within stride lengths has been observed to decrease with training^46^. Together, these observations indicate that variability within global mechanical behaviors may be a barometer for performance, skill, and fatigue in runners, but it has yet to be studied within elite runners or investigated in relation to the systemic spring-mass parameters across speeds.

As global mechanical parameters that are modeled by spring-mass dynamics and their respective levels of variability may have implications for running performance, the systemic characterization of the model’s behavior across a variety of speeds in a group of elite runners may reveal fundamental mechanistic insights that relate to expertise and performance in the sport. The goal of this study was to explore how spring-mass mechanics changed across a variety of training and racing speeds in elite middle distance runners and to compare those patterns to trained, but non-elite middle distance runners. The hypotheses were that all parameters would exhibit similar speed dependencies in both groups, that the elite runners would have distinct spring-mass characteristics independent of speed, and that the variation in the parameters across speeds would be lower in the elite runners.

## Methods

### Experimental data collection

#### Subjects

Ten elite-level male middle distance runners were recruited and enrolled in the study (inclusion criteria below). They were matched by 10 trained, but not elite-level male middle distance runners. The study was restricted to self-identified 1500 m/mile specialists. Given the high heterogeneity of training and physiological profiles within middle distance disciplines, we chose this athlete profile so as to select runners exposed to and familiar with a large spectrum of speeds in training^47^. Given the distinct nature of the subject population, this enrollment was subject to convenience sampling, and the target was based on similar studies of elite distance runners. A previous biomechanical study of elite distance runners found an average coefficient of variation of 8% among stride length, stride rate, swing time, flight time, and contact time^13^. Assuming this coefficient of variation in the observations and controlling for Type I error with α = 0.05, 9 subjects in each cohort would allow for detection of a 10% difference in means with statistical power of 1-β = 0.80^48^. Though this investigation sought to analyze the gait patterns of the runners with the mixed-effects linear regression modeling described below, this effect size approximation of the convenience sample nonetheless provided some context for the expected power of the study.

Inclusion criteria for the elite cohort of runners required that the subjects had achieved a sanctioned race performance in a long middle distance track event (1500 m or mile) equivalent to or greater than 1075 points per IAAF scoring tables in the current or previous competitive season (1500 m equivalent of 3:42.4 for males)^49^. Inclusion criteria for the trained cohort of middle distance runners required that they had achieved a sanctioned race performance equivalent to or greater than 465 but less than 1075 points in the current or previous competitive season (1500 m equivalent of 3:42.5 to 4:38.0)^49^. All participants were required to be free of lower limb injury at the time of testing and possess familiarity with treadmill running. The investigation was approved by the University of Michigan’s Institutional Review Board in accordance with the principles set forth in the Declaration of Helsinki, and all subjects provided written informed consent.

#### Running protocol

All subjects ran for 20 min at a self-selected pace on a treadmill as a warm-up. Following this warm-up, subjects ran at four submaximal running velocities for four minutes each, separated by a brief (30–45 s) pause. The elite cohort ran at 12 km/h, 14 km/h, 16 km/h, and 18 km/h, and the trained cohort ran at 10 km/h, 12 km/h, 14 km/h, and 16 km/h. These speeds represent typical training speeds for runners of this caliber^50^. Following these steady-state submaximal running bouts, the runners ran a series of six 30-s trials at interval-training and racing paces incrementing from 20 to 25 km/h in the elite cohort and 18 to 23 km/h in the trained cohort. Each trial was separated by 90 s of jogging at 11–12 km/h so that the full bout of six 30-s runs was continuous. The subjects ran the entire protocol in their preferred lightweight racing flats. Each 4 min trial was expected to capture 600–700 step cycles for each runner, and each 30 s trial was expected to capture 80–100 step cycles for each runner, totaling approximately 3000 steps per runner for analysis. This exceeded the 32–64 steps recommended as a minimum by Belli et al. to characterize mechanical parameter variability^44^.

#### Spatiotemporal measures

Contact time (t_c_) and flight time (t_f_) were recorded continuously throughout the running sessions at 100 Hz via a treadmill instrumented with a pressure plate (h/p/cosmos Quasar, h/p/cosmos Sports and Medical gmbh, Nussdorf-Traunstein, Germany). This system has demonstrated agreement and reliability in contact time measurement with a photoelectric timing system^51^. The platform had a sensing area of 1.36 × 0.64 cm with 10,240 sensors with detection thresholds of 1 N/cm^2^. The trials were recorded using Noraxon MyoMotion software (Noraxon USA, Scottsdale, AZ, USA), and the continuous data were exported for step cycle analysis in MatLab (MathWorks, Natick, MA, USA).

#### Spring-mass characteristics

The spring-mass parameters for each step were calculated using the method of Morin et al.^52^. Briefly, this modeled the vertical ground reaction force as a sinusoid with the subject’s body mass, m, and used the observed t_c_ and t_f_ to approximate the maximal vGRF, F_max_:

1 $${F}_{max}= mg\frac{\pi }{2}\left(\frac{{t}_{f}}{{t}_{c}}+1\right)$$

The center-of-mass’s absolute displacement during stance, Δy, was then modeled as:

2 $$\Delta y= \frac{{F}_{max}}{m}\frac{{{t}_{c}}^{2}}{{\pi }^{2}}-g\frac{{{t}_{c}}^{2}}{8}$$

Maximal leg compression of the spring during stance, ΔL, was approximated using the subject’s measured leg length, L_0_, and running speed, v, as:

3 $$\Delta L={L}_{0} -\sqrt{{{L}_{0}}^{2}-{\left(\frac{v{t}_{c}}{2}\right)}^{2}}+\Delta y$$

The impact angle of the spring, α, was approximated using L_0_, t_c_, and v^53^:

4 $$\alpha ={\mathit{cos}}^{-1}\left(\frac{v{t}_{c}}{2{L}_{0}}\right)$$

From these values, leg stiffness, k_leg_, was calculated as F_max_/ΔL, and vertical stiffness, k_vert_, was calculated as F_max_/Δy. Figure 1 illustrates the interpretation of these measures.

### Data analysis

Spatiotemporal measures (contact time (t_c_), flight time (t_f_), duty factor (DF), stride length (SL), and stride frequency (SF)) and traditional spring-mass measures (k_leg_, k_vert_, center-of-mass displacement (**Δ**y), leg compression (**Δ**L), and approximated maximal vertical force, F_max_) were calculated for each step captured. Within each trial at each speed, the coefficient of variation (CV) for each measure was calculated as σ/μ. Analysis of the measures across speeds was conducted using mixed-effect model linear regression, where the measure was treated as the response variable, the cohort (elite vs. trained) as a discrete fixed effect, and the speed as a continuous fixed effect with an interaction. Each subject was assigned a random effect intercept with a random slope corresponding to an individual speed-dependency:

5 $$y= {\beta }_{intercept}+{\beta }_{speed}+{\beta }_{cohort}+{\beta }_{speed}\times {\beta }_{cohort}+{\gamma }_{intercept}{+\gamma }_{speed}+\varepsilon$$

For the coefficient-of-variation models, the random slope was excluded, as the measures were aggregated as single observations at each speed for each subject. All predictors were centered prior to analysis, with the groups being assigned -0.5 and 0.5 for the trained and elite groups, respectively. For the linear mixed-effect models, the fixed effects were assessed for significance via Satterthwaite’s method. Statistical test criterion in all models used a Type I error control of α < 0.05. MatLab (2019a, MathWorks, Natick, MA, USA) was used for all data processing, and R (v3.6.2, R Foundation for Statistical Computing, Vienna, Austria) was used for all statistical analyses.

## Results

The subject characteristics are given in Table 1. A total of 70,812 steps were recorded. For each subject across the ten speeds, 3540 ± 157 steps were captured (mean ± s.d.), with 738 ± 60 steps for each of the four submaximal speeds, and 98 ± 30 steps for each of the six interval/racing speeds. For all fixed effects and interactions presented below, the values are described as the effect ± standard error.

**Table 1:** Elite and trained cohort characteristics.

Characteristic	Elite	Trained
Subjects (n)	10	10
Age (years)	27.7 ± 3.8	23.7 ± 4.0
Mass (kg)	70.4 ± 6.2	64.1 ± 3.6
Height (m)	1.82 ± 0.08	1.78 ± 0.06
Leg length (m)	0.956 ± 0.044	0.925 ± 0.049
IAAF best	1143 ± 50	768 ± 42
Mile best (min:s)	3:54.6 ± 3.9 s	4:27.4 ± 4.1 s
1500 m best (min:s)	3:37.4 ± 3.6 s	4:07.6 ± 3.7 s
1500 m speed (m/s)	6.90 ± 0.12	6.06 ± 0.09

Data are provided as mean ± standard deviation. IAAF Performance scores were calculated for the subject’s best 1500 m or mile performance, and the summary times or their event equivalent are provided for both the mile and 1500 m.

### Spatiotemporal measures

The results for the spatiotemporal measures are provided in Table 2. Each of the spatiotemporal measures had significant speed dependencies independent of cohort (all speed effects *p* < 0.001). However, the elite runners were more stable in their contact times across speeds than the trained runners, with their interaction on the speed being + 2 ± 0.4 ms per km/h (*p* < 0.001). The flight times of the elites were 15 ± 7 ms higher (*p* = 0.040) and also similarly changed less across speeds (− 1 ± 0.3 ms per km/h , *p* = 0.019). This resulted in duty factors that were lower in the elites—though the fixed effect estimate did not reach the threshold for significance (*p* = 0.059)—and less affected by speed than the trained runners (− 0.002 ± 0.001 per km/h, *p* = 0.016). As such, stride frequencies and stride lengths were ultimately not significantly different between groups.

**Table 2:** Spatiotemporal estimates with effects for cohort, speed, and the interactions.

	t_c_ (ms)	sem	*p* value	sig
Trained	237	3.2	–	
Elite	− 7	6.5	0.262	
Speed	− 8	0.2	< 0.001	***
Cohort × speed	2	0.4	< 0.001	***

	t_f_ (ms)	sem	*p* value	sig
Trained	132	3.3	–	
Elite	+ 15	6.7	0.040	*
Speed	3	0.3	< 0.001	***
Cohort × speed	− 1	0.6	0.019	*

	DF	sem	*p* value	sig
Trained	0.321	0.004	–	
Elite	− 0.017	0.008	0.059	
Speed	− 0.007	0.000	< 0.001	***
Cohort × speed	0.002	0.001	0.016	*

	SF (Hz)	sem	*p* value	sig
Trained	2.72	0.02	–	
Elite	− 0.06	0.05	0.254	
Speed	0.04	0.00	< 0.001	***
Cohort × speed	0.00	0.00	0.913	

	SL (m)	sem	*p* value	sig
Trained	1.42	0.015	–	
Elite	+ 0.03	0.030	0.289	
Speed	0.07	0.001	< 0.001	***
Cohort × speed	0.00	0.00	0.294	

The values provided for the trained cohort correspond to the model estimate at 14 km/h, with the elite difference, speed-relation (all subject change per km/h), and cohort-speed interaction presented below that. Estimated standard errors are provided for each effect^68^. Statistical significance of each effect is indicated as: *p < 0.05 and ***p < 0.001.

### Spring-mass measures

**Figure 2:**
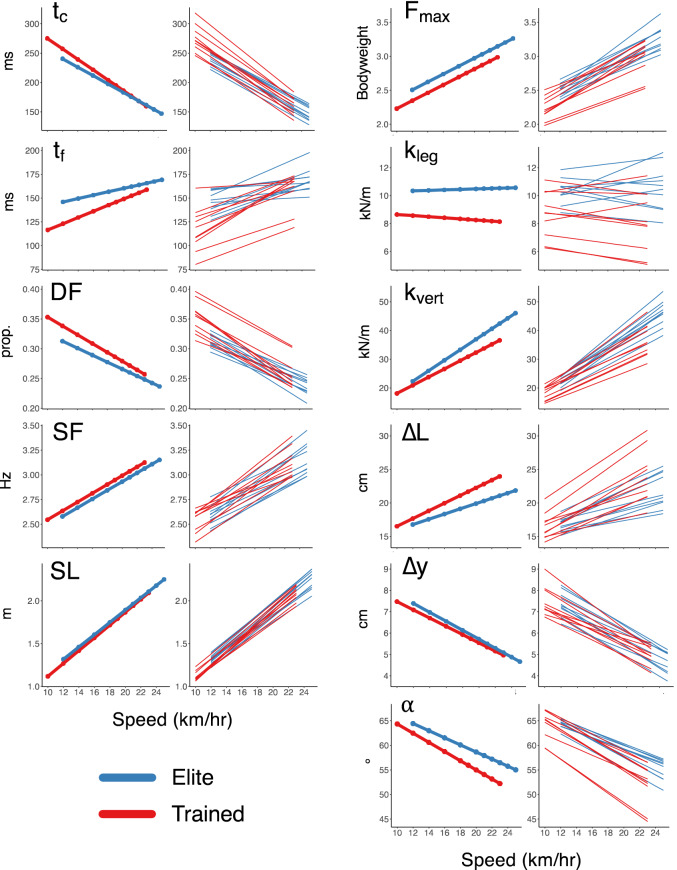
Spatiotemporal and spring-mass characteristics for elite and trained runners across speeds. Population (fixed, left) and individual (random, right) effects given for each measure.

**Figure 3:**
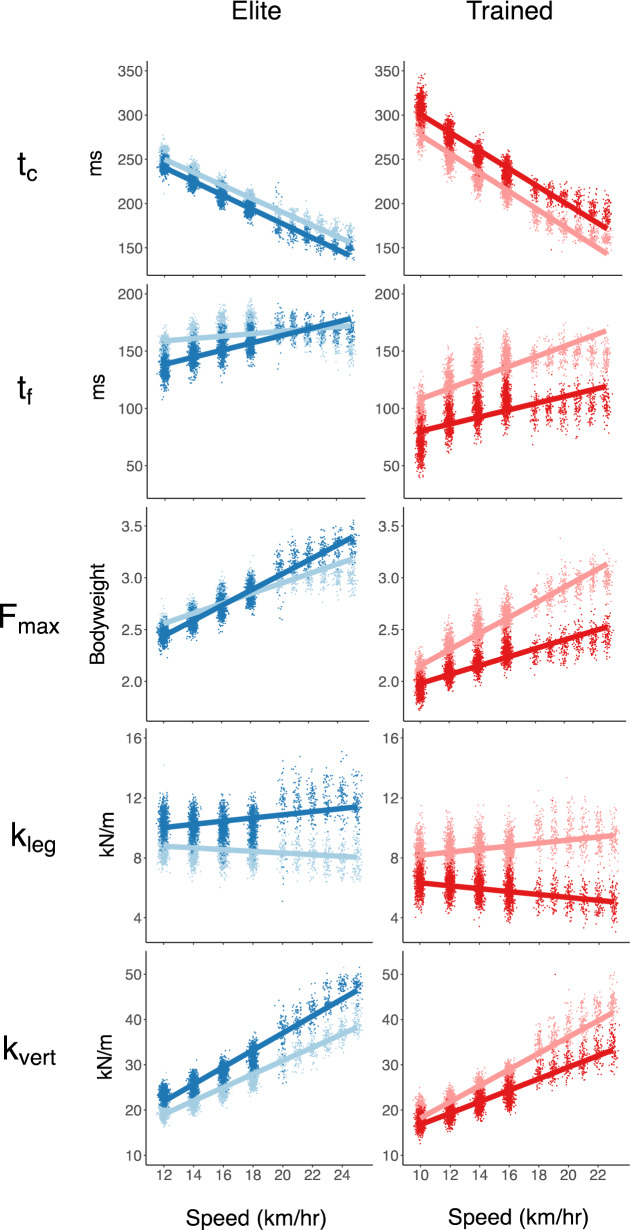
Select spring-mass characteristics across speeds for two representative subjects for the elite (left) and trained^67^ cohorts.

The results for the spring-mass characteristics are provided in Table 3. The approximated peak vertical forces were higher in the elite runners across speeds (+ 0.16 ± 0.07 BW, *p* = 0.045), while speed affected the approximated forces of both groups similarly. The elite runners had higher leg stiffnesses (+ 2.1 ± 0.7 kN/m, *p* = 0.007) across speeds, but neither cohort significantly altered their stiffnesses with speed. Similarly, the elite runners exhibited higher vertical stiffnesses (+ 3.6 ± 1.5 kN/m, *p* = 0.031). While both groups increased their vertical stiffnesses across speeds (6.6 ± 0.1 kN/m per km/h, *p* < 0.001), the elite runners did more so (0.40 ± 0.1 kN/m per km/h, *p* = 0.006). However, their vertical displacements during stance were similar, with both groups decreasing vertical motion at faster speeds equivalently (− 0.2 ± 0.02 cm per km/h, *p* < 0.001). Both groups increased their leg compression at faster speeds (0.5 ± 0.03 cm per km/h, *p* < 0.001), but the elite runners’ compressions were less affected by speed increases (− 0.2 ± 0.07 cm per km/h, *p* = 0.024). The elite runners also ran with larger impact angles—i.e., with more upright, vertically-oriented springs—across speeds (+ 3.1 ± 1.2°, *p* = 0.020). Both groups decreased their impact angles at faster speeds (− 0.83 ± 0.03° per km/h, *p* < 0.001), but in tandem with the leg compression observations, the elites exhibited less of a decrease in their angles at faster speeds (+ 0.21 ± 0.06° per km/h, *p* = 0.004). The spatiotemporal and spring-mass trends are presented in Fig. 2, where within each measure, the population effects (fixed) and individual effects (random) are presented on the left and right, respectively. The full set of observations for two representative subjects from each cohort are shown (Fig. 3) for contact time, flight time, vertical force, leg stiffness, and vertical stiffness.

**Table 3:** Spring-mass characteristics with effects for cohort, speed, and the interactions.

	F_max_ (BW)	sem	*p *value	sig
Trained	2.46	0.037	–	
Elite	+ 0.16	0.074	0.045	*
Speed	0.06	0.003	< 0.001	***
Cohort × speed	0.00	0.006	0.997	

	kl_eg_ (kN/m)	sem	*p *value	
Trained	8.39	0.344	–	
Elite	2.09	0.689	0.007	**
Speed	− 0.01	0.021	0.607	
Cohort × speed	0.06	0.042	0.197	

	k_vert_ (kN/m)	sem	*p *value	
Trained	23.07	0.769	–	
Elite	+ 3.60	1.538	0.031	*
Speed	1.62	0.065	< 0.001	***
Cohort × speed	0.40	0.130	0.006	**

	ΔL (cm)	sem	*p* value	
Trained	19.12	0.536	–	
Elite	–1.89	1.072	0.095	
Speed	0.48	0.037	< 0.001	***
Cohort × speed	–0.18	0.074	0.024	*

	Δy (cm)	sem	*p* value	
Trained	6.73	0.108	–	
Elite	+ 0.21	0.216	0.340	
Speed	− 0.20	0.010	< 0.001	***
Cohort × speed	− 0.02	0.019	0.409	

	α (°)	sem	*p* value	
Trained	63.50	0.613	–	
Elite	+ 3.12	1.225	0.020	*
Speed	− 0.83	0.032	< 0.001	***
Cohort × speed	0.21	0.064	0.004	**

The values provided for the trained cohort correspond to the model estimate at 14 km/h, with the elite difference, speed-relation (all subject change per km/h), and cohort-speed interaction presented below that. Estimated standard errors are provided for each effect^68^. Statistical significance of each effect is indicated as: *p < 0.05, **p < 0.01, and ***p < 0.001.

### Coefficient of variation

The coefficients of variation for the spatiotemporal measures are presented similarly (Table 4). Both cohorts had greater variation in contact time at faster speeds (+ 0.05 ± 0.02% per km/h, *p* = 0.014), but there were no differences between groups. The elite runners had significantly lower variations in their flight times (− 1.3 ± 0.4%, *p* = 0.006). Both groups decreased variation at faster speeds (− 0.24 ± 0.02% per km/h, *p* < 0.001), but the elite runners were less affected by speed increases (+ 0.19 ± 0.04% per km/h, *p* < 0.001). Ultimately, variations in duty factors were similar between groups and unaffected by speed, but stride frequency variation was significantly lower in the elite runners (− 0.3 ± 0.1%, *p* = 0.017) and higher across speeds in both groups (+ 0.04 ± 0.01%, *p* < 0.001). Similarly, stride length variability was lower in the elite runners (− 0.3 ± 0.1%, *p* = 0.019), and both groups increased stride length variability at faster speeds (+ 0.03 ± 0.01%, *p* < 0.001).

**Table 4:** Coefficients of variation (expressed as a %) for spatiotemporal measures with effects for cohort, speed, and the interactions.

	t_c_ (ms)	sem	*p* value	sig
Trained	3.35	0.12	–	
Elite	− 0.13	0.24	0.573	
Speed	0.05	0.02	0.014	*
Cohort × speed	0.01	0.04	0.881	

	t_f_ (ms)	sem	*p* value	
Trained	7.34	0.21	–	
Elite	− 1.32	0.43	0.006	**
Speed	− 0.24	0.02	< 0.001	***
Cohort × speed	0.19	0.04	< 0.001	***

	DF	sem	*p* value	
Trained	3.30	0.103	–	
Elite	− 0.10	0.205	0.631	
Speed	0.00	0.015	0.957	
Cohort × speed	0.02	0.029	0.528	

	SF (Hz)	sem	*p* value	
Trained	2.40	0.06	–	
Elite	− 0.31	0.12	0.017	*
Speed	0.04	0.01	< 0.001	***
Cohort × speed	0.01	0.02	0.595	

	SL (m)	sem	*p* value	
Trained	2.39	0.06	–	
Elite	− 0.30	0.12	0.019	*
Speed	0.03	0.01	< 0.001	***
Cohort × speed	0.01	0.01	0.564	

The values provided for the trained cohort correspond to the model estimate at 14 km/h, with the elite difference, speed-relation (all subject change per km/h), and cohort-speed interaction presented below that. Estimated standard errors are provided for each effect^68^. Statistical significance of each effect is indicated as: *p < 0.05, **p < 0.01, and ***p < 0.001.

**Figure 4:**
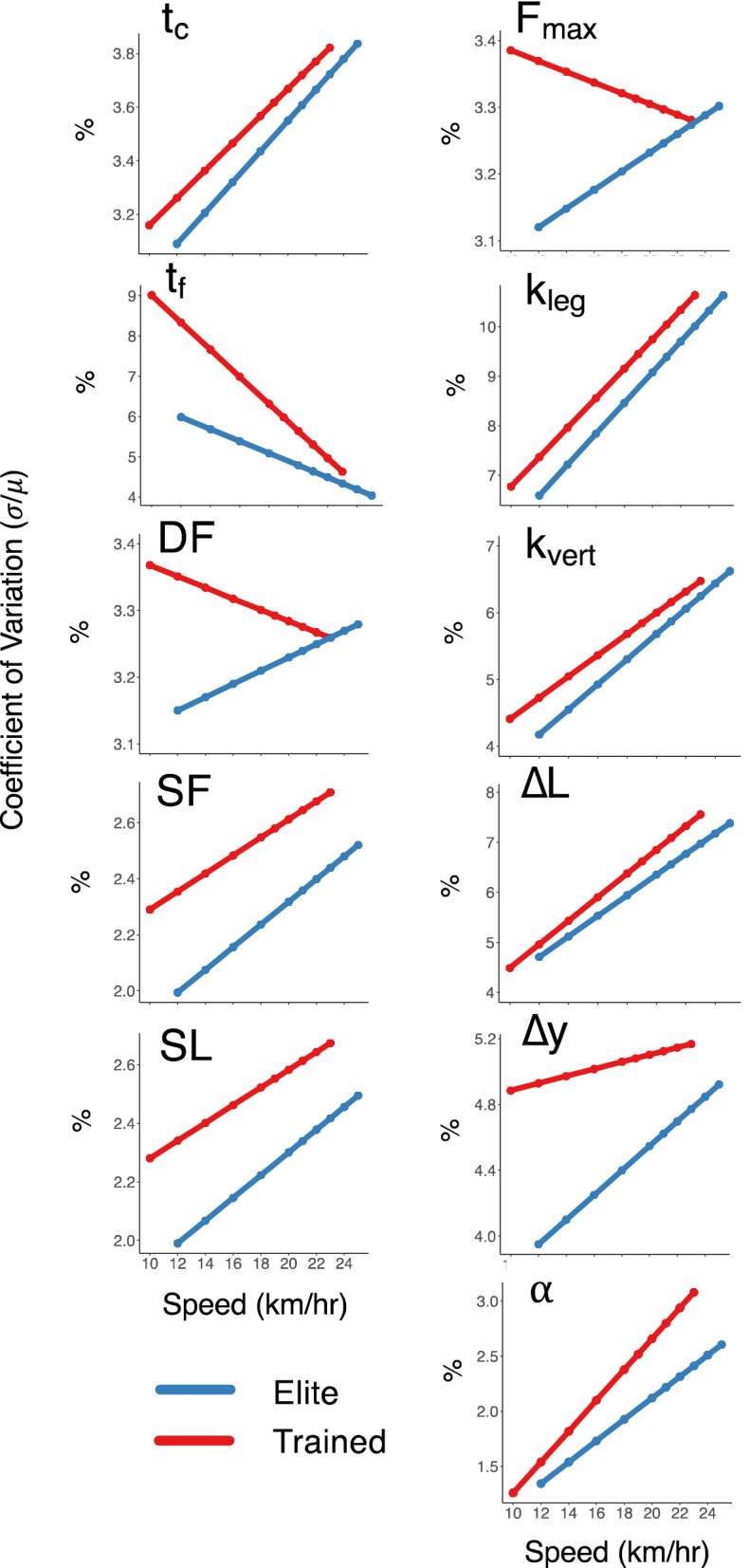
Coefficient of variations at each speed for the spatiotemporal and spring-mass characteristics of the elite and trained runners.

The coefficients of variation for the spring-mass characteristics (Table 5) indicated that the variations in peak vertical forces were equal between groups and did not change with speed. Leg stiffness and vertical stiffness variation was similarly similar between both groups, but both measures saw significant increases across speeds of 0.30 ± 0.06% per km/h (*p* < 0.001) and 0.17 ± 0.04% per km/h (*p* < 0.001), respectively. Leg compression variation was similarly equal between groups and also increased with speed 0.22 ± 0.04% per km/h (*p* < 0.001). The variation in vertical displacement was lower in the elite runners across speeds (− 0.7 ± 0.3%, *p* = 0.018), and both groups exhibited modest increases with speed (+ 0.05 ± 0.02% per km/h, *p* = 0.008). The elite runners also had less variation in their impact angles across speeds (− 0.4 ± 0.2%, *p* = 0.026), and both groups increased their angle variation at faster speeds (0.12 ± 0.02% per km/h, *p* < 0.001). The group trends for the variation patterns in each measure are presented in Fig. 4.

**Table 5:** Coefficients of variation (expressed as a %) for spring-mass characteristics with effects for cohort, speed, and the interactions.

	F_max_ (BW)	sem	*p* value	sig
Trained	3.31	0.10	–	
Elite	− 0.13	0.21	0.542	
Speed	0.00	0.01	0.836	
Cohort × speed	0.02	0.03	0.441	

	k_leg_ (kN/m)	sem	*p* value	
Trained	7.94	0.26	–	
Elite	− 0.70	0.52	0.195	
Speed	0.30	0.06	< 0.001	***
Cohort × speed	0.01	0.12	0.909	

	k_vert_ (kN/m)	sem	*p* value	
Trained	4.99	0.17	–	
Elite	− 0.39	0.35	0.274	
Speed	0.17	0.04	< 0.001	***
Cohort × speed	0.03	0.07	0.683	

	ΔL (cm)	sem	*p* value	
Trained	5.49	0.21	–	
Elite	− 0.42	0.41	0.322	
Speed	0.22	0.04	< 0.001	***
Cohort × speed	− 0.03	0.08	0.706	

	Δy (cm)	sem	*p* value	
Trained	4.88	0.13	–	
Elite	− 0.69	0.27	0.018	*
Speed	0.05	0.02	0.008	**
Cohort × speed	0.05	0.04	0.139	

	α (°)	sem	*p* value	
Trained	1.89	0.09		
Elite	− 0.43	0.18	0.026	*
Speed	0.12	0.02	< 0.001	***
Cohort × speed	− 0.04	0.03	0.189	

The values provided for the trained cohort correspond to the model estimate at 14 km/h with the elite difference, speed-relation (all subject change per km/h), and cohort-speed interaction presented below that. Estimated standard errors are provided for each effect^68^. Statistical significance of each effect is indicated as: * *p* < 0.05, ** *p* < 0.01, and *** *p* < 0.001.

## Discussion

### Summary

Across running speeds, elite middle distance runners generally ran as stiffer mechanical systems than their highly-trained but non-elite counterparts, and they tended to adjust their mechanics differently in response to speed changes. The spatiotemporal characteristics of both groups were strongly speed-dependent, but the flight times and corresponding duty factors were less affected in the elite runners. The maximal vertical forces were similarly speed-dependent in both groups but were consistently higher in the elite runners. Spring-mass analyses demonstrated that the elites had higher leg and vertical stiffnesses, and they augmented their vertical stiffnesses more at faster speeds. The elite runners also demonstrated less variability in some of the mechanical parameters, with their fight times, stride lengths, stride frequencies, impact angles, and vertical displacements being less variable across speeds than that of the trained runners. These results suggested that elite middle distance runners exhibited systemic mechanical patterns that distinguish them from other highly-trained middle distance runners.

### Spatiotemporal measures

As hypothesized, all of the spatiotemporal measures exhibited strong relations with speed. In both cohorts, contact time decreased substantially across speeds, and flight time increased modestly, resulting in a duty factor that progressively decreased across speeds. In the elite runners, the speed relation was significantly smaller, where their ground contacts were shorter in the submaximal speeds but similar at the faster speeds. Their flight times were 11% greater on average across speeds, and their speed-dependent increase was smaller, with their flight times at lower speeds being relatively longer. This resulted in duty factors that were lower and similarly more stable across the range of speeds. This was consistent with both the observations of Leskinen and colleagues, who observed similar contact times and stride frequencies between national-class and elite-level 1500-m runners during a race (~ 23 km/h)^15^, and Folland and colleagues, who observed a significant negative correlation between performance and both ground contact time and duty factor at lower speeds (10–12 km/h) in a large group of middle- and long-distance runners^23^. This was also similar to the observations of Williams and Cavanagh, who observed a modest correlation between contact time at 12.8 km/h and a 10-km time trial performance^10^, as well as Nummela and colleagues, who observed a correlation between submaximal oxygen consumption and contact time that diminished with speed^24^. The contact and flight time trends resulted in stride lengths and frequencies that were still equivocal between groups, and similarly exhibited strong speed-dependent increases. This was consistent with previous observations of speed relations within these parameters^54^, as well as their independence from ability or performance^10,13,23^.

### Spring-mass behavior

The findings here supported the hypothesis for distinct spring-mass behavior among the elite runners. Their estimated peak vertical forces were 6% higher relative to their body weight, and this, coupled with the temporal differences, resulted in higher stiffness measures. Their leg and vertical stiffnesses were 25% and 16% higher on average across speeds, and while both groups increased vertical stiffness with speed, the elites did so more substantially (5.2 vs. 3.6 kN/m per km/h). Leg stiffness was independent of speed in both groups, consistent with previous observations in other runners^52,53^ and across animal species^31^. Farley and colleagues posited that runners maintain leg stiffness across speeds by increasing their sweep angle through stance at faster speeds—i.e., decreasing their impact angle. With greater vertical forces, they thereby compress their effective spring more. The decreased impact angle, greater force, and greater speed interact to maintain a consistent center-of-mass trajectory through stance across speeds, which with the greater force, then ultimately increases vertical stiffness and decreases the contact time^31^. This was consistent with our observations, where the impact angles among both groups decreased by 0.8° per km/h as the subjects ran faster. The resulting leg compressions increased by 0.5 cm per km/h, while the vertical displacement only decreased by 0.2 cm per km/h.

These patterns together suggested that the elite runners may be better exploiting the elastic mechanisms underpinning spring-mass dynamics. Their stiffer effective springs, coupled with greater vertical forces, steeper impact angles, and more speed-sensitive vertical stiffnesses, suggested that they may be recycling kinetic energy more efficiently throughout the gait cycle. The patterns observed here indicated more vertical orientation of their effective elastic mechanisms, evidenced by their larger impact angles across speeds (+ 3.1°) that declined less at faster speeds, implying shorter braking and propulsive periods. As horizontal force generation is metabolically much more expensive than vertical force generation^55^, this propensity to orient their spring-mass dynamics more vertically may explain their greater performance capacity. Furthermore, steeper impact angles are characteristic of spring-mass systems that can run passively with greater stability^56^. The exhibition of shorter contact times and longer flight times at lower speeds may also be a reflection of more efficient use of these elastic storage and return mechanisms. A more rapid and forceful stretch of musculoskeletal tendinous structures has been demonstrated to improve the elastic efficiency of those structures^57,58^. The contribution of this recycled elastic energy to the cost of running has been shown to increase with speeds^59^, and this elastic, spring-like behavior has been proposed as the predominant means that mediates the energetic efficiency of running across speeds and species^60,61^. For the elite runners, their quicker interactions with the ground via their stiffer effective systems may be better leveraging and orchestrating the elastic structures of own their bodies to recycle the gravitational potential energy of flight more efficiently.

It may be that elite middle distance runners have more robust or refined mechanisms to exploit these advantages. That this behavior was distinctly persistent at lower speeds may further indicate a “mastery” of these mechanisms. However, whether this is a product of their innate ability or their high volume of accumulated lifetime training could not be inferred from this investigation. It is more likely that the characteristics described above are not products of one or the other attributes exclusively, but rather that their innate characteristics facilitated their exceptional training to further refine and exploit those characteristics. That is, the interaction of nature and nurture may have given rise to their emergent, elite ability.

### Parameter variability

The elite runners further demonstrated lower variability in several of the measures across speeds, including flight times, stride frequencies, stride lengths, impact angles, and their vertical displacements during stance. This was consistent with previous investigations that explored pattern variability in relation to running economy. In trained runners, stride time and vertical oscillation have been associated with better economy^44,45^ and experience level^43^, similar to the stride frequency, vertical displacement, impact angle, and flight time patterns observed here. Furthermore, Slawinski and colleagues observed stride frequency variability to decrease after a structured training program in already competitive runners^46^. This decrease was associated with an improvement in running economy and velocity at VO_2max_ despite maximal aerobic capacity remaining unchanged, suggesting that the decreased variability was related to the improvement in efficiency. Spatiotemporal variability has also been observed to be lower among elite racewalkers^42^. These observations coupled with our findings support the notion that variability is associated with skill and expertise. However, the “optimal” amount is still unknown, and it is likely highly individualized. The elite runners here exhibited more control over those parameters across speeds, but it is certainly plausible that further decreasing the variability would be detrimental, where the motor control required could come at an energetic or structural cost. Elite triple jumpers demonstrated a parabolic curve with relation to coordination variability and performance, suggesting that optimality was not simply a minimization exercise^39^. It would be insightful to explore these patterns longitudinally within elite runners in a fashion similar to the investigation of Slawinski and colleagues^46^. Would these patterns change with performance level or injury status? It is also curious that distinct trends emerged within the spatiotemporal measures, yet they were largely absent among the spring-mass parameters. This may indicate that the spring-mass dynamics are an emergent phenomena that are mediated by the spatiotemporal dynamics, with these spatiotemporal adjustments interacting with each other in more or less variable ways to produce a consistent bounce for a given speed. This would indicate that these control strategies are better regulated in the elite runners with less apparent step-to-step adjustment occurring.

### Methodological advantages

Aside from the mechanical observations, a secondary goal of the study was to demonstrate the methodological value of a mixed-model experimental design. Here, we studied these patterns not only between groups, but across speeds and within individuals. One notable observation was the relative homogeneity of patterns within the elite runners as compared to the trained cohort that presented across parameters. Many of the findings observed here may not have emerged if observations were made at a single speed, as the nuanced differences and interactions only emerged across speeds and with prolonged observation within individuals. It was notable that among the elite runners, most demonstrated relative linearity across speeds within most of the parameters, while some of the trained runners had nonlinear shifts at the faster speeds. This might be indicative of distinct mechanical strategies for submaximal running and sprinting, whereas the elite runners demonstrated a greater “fluency” across speeds. We provided a supplementary analysis (Appendix A) modeling each variable with a quadratic dependency on speed following a suggestion of nonlinearity in the raw data as seen in Fig. 3. Most of the variables maintained a dominantly linear trend, but some in the trained cohort did exhibit strong nonlinearity in their flight times and their corresponding duty factors. While this phenomenon warrants further investigation, it is suggestive that further insights that may be afforded by such an analysis.

**Figure 5:**
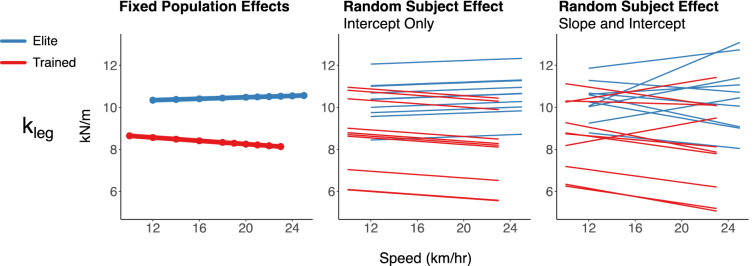
Leg stiffness estimates with the fixed effects model and two different random effects models.

Furthermore, as these experimental approaches gain traction and become standard-of-practice in biomechanical investigations, investigators should take great care in formulation of their analytical models. As an example, our mixed-effect models assigned not only a random intercept to each individual, but also a random slope across speeds, assuming that the speed-dependent changes would have an individualized pattern beyond that which was characteristic of the group (Figs. 2, 3). Had we simply used a random intercept model for each subject, it would have appeared that leg stiffness was not only significantly different between groups, but that there was a small, significant speed dependency and interaction. Both effects maintain similar values to what is reported in Table 3 (which were similarly small in magnitude), but their estimated standard errors were drastically reduced due to the apparent increase in degrees of freedom. By adding the random slope, the statistical significance of those marginal effects disappear, but the fit of the model significantly improves because the variance is better explained by the individualized response. The model’s Akaike information criterion decreases from 197,299 to 189,857 with the addition of the random slope term for speed, indicating significantly better fit (Likelihood-Ratio Chi-squared (2) = 7716.1, *p* < 0.001). This difference between the appearance of the effects between the two models is displayed in Fig. 5. For the variability analyses, we were restricted to using random intercept models, as the variation measures were aggregate values within a speed for each individual. As such, there were not repeated measures within individuals within speeds. Given the value demonstrated by random slope models for speed dependencies, it will be an interesting future line of investigation to aggregate groups of steps within a speed to further characterize these patterns with more individualized speed-dependencies modeled.

### Limitations

When interpreting the findings of this investigation, consideration must be given with respect to several aspects of the design. The first is that the spring-mass parameters reported here were calculated via the speed, contact time, and flights time as proposed by Morin et al.^52^. While this method has been validated against traditional kinetic methods and has demonstrated excellent agreement with those values^62,63^, measures of maximal vertical force and center-of-mass displacement during stance reported here were nonetheless approximations using these temporal and dynamic relations. Furthermore, the temporal characteristics were measured at 100 Hz, which was low compared to gold-standard kinetic measurements. Some investigators analyzing spring-mass or spatiotemporal dynamics have used rates as low as 80 Hz^64^ and even 50 Hz^29,46^, and they mitigated the low sensitivity by aggregating multiple observations. The system used here has demonstrated excellent agreement and reliability with a commonly used photoelectric timing system^51^, but we anticipated that the sensitivity of the measures would be similarly reduced compared to systems with higher resolution. This would have been problematic in analyzing a limited number of steps, but it was increasingly resolved as more observations were taken. As one of the strengths of the study was the large number of observations captured for each subject, we were able to detect trends between groups and across speeds with a large number of data points for each subject at each condition. However, we cannot rule out errors or detection failure on any effects or trends due to the sensitivity reduction.

Another aspect to be considered is that the observations were limited to that of male runners. In studying elite female distance runners, Williams and colleagues found that the elite females generated greater peak vertical forces with shorter ground contacts as compared to non-elite females at the same speed. However, when compared to a group of previously-studied elite males at the same speed, the elite female runners exhibited subtle differences in hip kinematics, longer relative stride lengths, and smaller relative vertical oscillations^11^. These findings would suggest that a population of elite female middle distance runners may also exhibit global mechanical patterns distinct from non-elite counterparts, similar to the observations between the male cohorts here. However, their findings would also suggest that the nature of the trends presented here may not necessarily extrapolate analogously for all variables onto a population of high-performing females. As such, a similar characterization of these patterns across speeds and abilities within female middle distance runners would be a necessary contribution to fully explore the persistence of these phenomena.

Finally, when interpreting the results within different contexts, it should be remembered that these observations were collected during treadmill running as opposed to overground running. This afforded the continuous collection, and the participants were required to be experienced with treadmill running. However, some of the runners may have exhibited different behavior in this different context^65^.

## Conclusion

Elite middle distance runners exhibited distinct systemic spring-mass behavior across a wide spectrum of running speeds as compared to a group of competitive, yet sub-elite counterparts. We examined these global mechanical characteristics in an elite cohort of 1500 m runners that included Olympians, National Champions, and NCAA All-Americans, and we compared the observations to those from a trained cohort that included 1500 m runners who were regionally competitive NCAA and university club-level athletes. Despite the high ability and training status of both groups, the elite runners distinguished themselves across a number of spatiotemporal and spring-mass parameters, and those differences were further mediated across speeds. The elite group generally ran with longer flight phases and as stiffer systems, producing greater vertical forces and higher leg and vertical stiffnesses. Furthermore, their spatiotemporal patterns were less variable across speeds. These distinct systemic patterns and interactions across running speeds may be related to some combination of superior ability, training characteristics, or inherent physiological attributes. The findings presented a profile of global mechanics in top-level middle distance runners, which may serve as a reference for future investigations or for coaches and athletes conducting performance assessments. Furthermore, it highlighted the importance of analyzing running mechanics both across and within a breadth of speeds for individuals.

## Supplementary Information

Supplementary Information.
