# Supplementary material for: Bouncing behavior of sub-four minute milers
Source: Sci Rep. 2021 May 18;11:10501. doi: 10.1038/s41598-021-89858-1 (PMC8131362; doi:10.1038/s41598-021-89858-1)

Supplementary Material

1 S1 – Quadratic Models

For each of the variables investigated, we have included an analysis that also gives a coefficient for a quadratic expression of the speed-dependency, where the squared speed term has both a fixed effect with its own interaction with the grouping and a random effect for each subject:

$$y = \beta_{intercept} + \beta_{speed} + \beta_{cohort} + \beta_{speed2} + \beta_{speed} \times \beta_{cohort} + \beta_{speed2} \times \beta_{cohort} + \gamma_{intercept} + \gamma_{speed} + \gamma_{speed2} + \varepsilon \quad (1)$$

The rationale for this additional analyses is that there is a suggestion of slight curvature in the raw data as seen in Figure 3. The results are provided in Table S1 and Table S2 and are illustrated in Figure S1.

Many of the variables follow the same patterns observed in the linear models presented in Table 2, Table 3, and Figure 2. In all of the variables with significant speed dependencies, both the linear and quadratic terms were found to be significant, though the magnitude of the curvature in most was mild. The notable exceptions were in the flight times and duty factors, where both groups exhibited more substantial nonlinearity at faster speeds. The flight times effectively leveled off with downward concavity, which manifested, albeit with a lesser magnitude, in the upward concavity of the duty factor trends. The group interactions with the quadratic terms were also significant with these variables, with earlier and more substantial curvature in the trained group, with some even decreasing their flight times at the fastest speeds. This further suggests that some individuals adopted a discrete systemic biomechanical pattern at the fastest speeds, possibly switching to distinct sprint-oriented mechanics. This “switch” was less apparent or absent among the elite runners, who maintained their trends more consistently across speeds. One final small distinction was observed in the leg stiffness models, where the linear speed coefficient was insignificant, consistent with the linear models, but the quadratic term was significant, indicating very mild upward concavity. This appeared to be driven by the elite group, where the interaction on the quadratic term approached significance ($p = 0.08$). As illustrated in Figure S1, the elite runners seem to have very mild minima near 18 km/hr. Though the magnitude of this is small, it suggests that their systems may be “stiffest” at the slowest and fastest speeds.

The quadratic fit is one possible approach to model nonlinearity–a matter which future research should explore in more depth. We focused the primary analyses in the text on the linear model to avoid overfitting, as the number of parameters begins to grow and larger samples may be needed to better understand subtle nonlinearities.

	**t_c (ms)**	**sem**	**p-value**		**effect (p)**
**Trained**	235.74	3.29	-		0.330
**Elite**	-6.59	6.58	0.330		
**Speed**	-7.42	0.18	<0.001	✻✻✻	<0.001
**Speed^2**	0.25	0.02	<0.001	✻✻✻	
**Cohort x Speed**	0.39	0.36	0.286		0.001
**Cohort x Speed^2**	-0.15	0.04	0.001	✻✻✻	

	**t_a (ms)**	**sem**	**p-value**		**effect (p)**
**Trained**	135.48	3.52	-		0.056
**Elite**	14.39	7.04	0.056		
**Speed**	1.55	0.29	<0.001	✻✻✻	<0.001
**Speed^2**	-0.43	0.03	<0.001	✻✻✻	
**Cohort x Speed**	0.61	0.59	0.316		0.030
**Cohort x Speed^2**	0.17	0.06	0.010	✻✻	

	**DF**	**sem**	**p-value**		**effect (p)**
**Trained**	0.32	0.004	-		0.083
**Elite**	-0.02	0.009	0.083		
**Speed**	-0.01	0.000	<0.001	✻✻✻	<0.001
**Speed^2**	0.00	0.000	<0.001	✻✻✻	
**Cohort x Speed**	0.00	0.001	0.397		0.006
**Cohort x Speed^2**	0.00	0.000	0.002	✻✻✻	

	**SF (Hz)**	**sem**	**p-value**		**effect (p)**
**Trained**	2.70	0.026	-		0.218
**Elite**	-0.07	0.051	0.218		
**Speed**	0.05	0.002	<0.001	✻✻✻	<0.001
**Speed^2**	0.00	0.000	<0.001	✻✻✻	
**Cohort x Speed**	-0.01	0.005	0.067		0.150
**Cohort x Speed^2**	0.00	0.000	0.748		

	**SL (m)**	**sem**	**p-value**		**effect (p)**
**Trained**	1.44	0.016	-		0.201
**Elite**	0.04	0.031	0.201		
**Speed**	0.07	0.002	<0.001	✻✻✻	<0.001
**Speed^2**	0.00	0.000	<0.001	✻✻✻	
**Cohort x Speed**	0.01	0.003	0.048	✻	0.090
**Cohort x Speed^2**	0.00	0.000	0.925		

Table S1 Spatiotemporal estimates for the groups with effects for group, speed, and the interactions. The values provided for the trained cohort correspond to the model estimate at 14 km/hr. Estimated standard errors are provided for each effect (sem). Statistical significance of each effect is indicated as: ✻ p <0.05 and ✻✻✻ p<0.001, and the p-value for each aggregate factor and interaction (linear and quadratic term together)

	F_{max} (BW)	sem	p-value		
**Trained**	2.49	0.039	-		0.061
**Elite**	0.16	0.078	0.061		
**Speed**	0.05	0.003	<0.001	✻✻✻	<0.001
**Speed^2**	0.00	0.000	<0.001	✻✻✻	
**Cohort x Speed**	0.01	0.006	0.084		0.100
**Cohort x Speed^2**	0.00	0.001	0.101		

	k_{leg} (kN/m)	sem	p-value		factor (p)
**Trained**	8.46	0.347	-		0.011
**Elite**	1.97	0.693	0.011	✻	
**Speed**	-0.01	0.022	0.739		0.024
**Speed^2**	0.01	0.002	0.009	✻✻	
**Cohort x Speed**	0.05	0.044	0.316		0.180
**Cohort x Speed^2**	0.01	0.004	0.088		

	k_{vert} (kN/m)	sem	p-value		factor (p)
**Trained**	23.20	0.752	-		0.051
**Elite**	3.15	1.505	0.051		
**Speed**	1.71	0.068	<0.001	✻✻✻	<0.001
**Speed^2**	0.06	0.005	<0.001	✻✻✻	
**Cohort x Speed**	0.20	0.135	0.162		0.154
**Cohort x Speed^2**	0.02	0.009	0.057		

	ΔL (cm)	sem	p-value		factor (p)
**Trained**	19.10	0.546	-		0.129
**Elite**	-1.74	1.093	0.129		
**Speed**	0.43	0.037	<0.001	✻✻✻	<0.001
**Speed^2**	-0.03	0.003	<0.001	✻✻✻	
**Cohort x Speed**	-0.08	0.073	0.303		0.556
**Cohort x Speed^2**	0.00	0.006	0.572		

	Δy (cm)	sem	p-value		factor (p)
**Trained**	6.80	0.112			0.409
**Elite**	0.19	0.224	0.409		
**Speed**	-0.22	0.010	<0.001	✻✻✻	<0.001
**Speed^2**	-0.01	0.001	<0.001	✻✻✻	
**Cohort x Speed**	0.02	0.019	0.374		0.061
**Cohort x Speed^2**	0.00	0.002	0.022	✻	

Table S2 Spring-mass characteristics for the groups with effects for group, speed, and the interactions. The values provided for the trained cohort correspond to the model estimate at 14 km/hr. Estimated standard errors are provided for each effect (sem). Statistical significance of each effect is indicated as: ✻ $p < 0.05$, ✻✻ $p < 0.01$, and ✻✻✻ $p < 0.001$

Figure S1: Spatiotemporal and spring-mass characteristics for elite and trained runners across speeds. Population (fixed, left) and individual (random, right) effects given for each measure

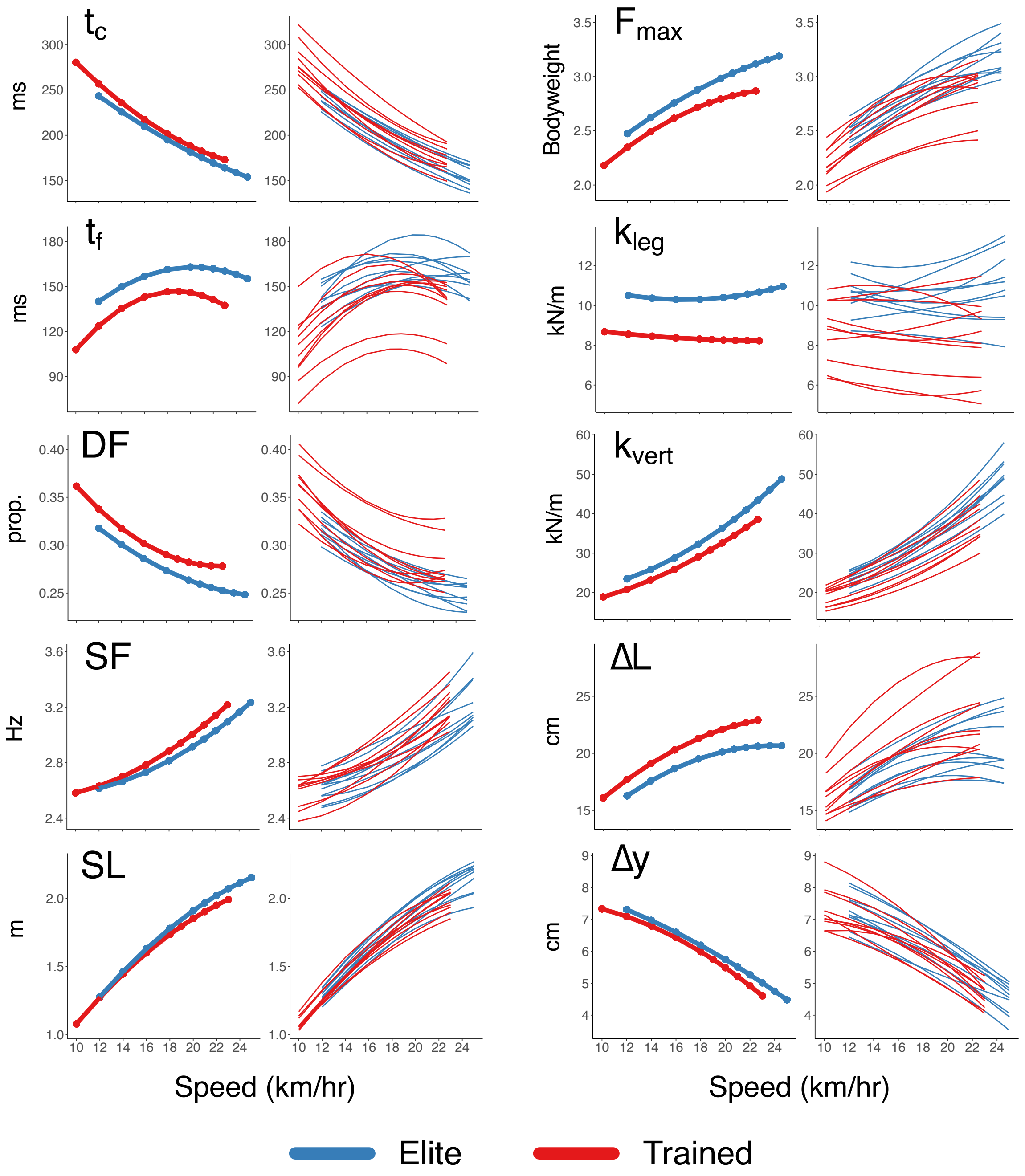

Supplement: Supplementary file 1 — Supplementary Information. [file 41598_2021_89858_MOESM1_ESM.pdf]
